# Response to: an exploratory cross-sectional study on the relationship between dispositional mindfulness and empathy in undergraduate medical students

**DOI:** 10.1080/10872981.2020.1826112

**Published:** 2020-09-22

**Authors:** Sithhipratha Arulrajan, Nazifa Ullah, Pranavan Pavanerathan

**Affiliations:** University College London Medical School, London, UK

**Keywords:** Dispositional mindfulness, medical education, empathy, medical students, mindfulness training

We were intrigued by the study by Ardenghi et al. [1] which explored the association between dispositional mindfulness (DM) and empathy, concluding with the need to incorporate facets of DM into the medical curriculum. As British medical students, we share the view that this is a much neglected concept in healthcare education and would like to provide recommendations based on our experiences on how DM can be integrated into medical education.

The importance of mindfulness training for clinicians in the UK was highlighted by the General Medical Council (GMC), which recognised its value in increasing well-being and resilience to stress, and consequently empathy [2]. In recognition of this our medical school, University College London (UCL), provided students with the opportunity to attend a new initiative known as Schwartz Centre Rounds (SCR) once a term. These rounds provide an opportunity for students and clinicians to meet together to discuss the emotional and social aspects of clinical cases. Such models of self-reflection endeavour to encourage participants to examine past experiences, highlight learning points and use these lessons as a framework to guide future circumstances [3]. We personally found that these sessions were beneficial as it encouraged us to explore our emotions in a non-judgemental space. Similar opinions were also collated from students who attended pilot SCR at UCL, with participants recognising the value that these sessions had in creating a culture of openness and a sense of community [4]. In fact, 82% of students believed that SCR gave them a better insight into others’ emotions and patient care [3]. Such studies depict the potential SCR have for improving indicators of DM and consequently empathy, and we would encourage further research into integrating SCR within medical curricula.

In further striving to implement mindfulness training amongst medical students, UCL has adopted other methods of reflection into their curriculum. For example, there is a requirement to complete reflective essays throughout our time at medical school. These essays explore topics such as ‘the patient you will never forget’ and ‘a time I worked in a team’. These exercises encouraged us to externalise our feelings about a clinical situation, evaluate our emotions and create an action plan to encourage or discourage the scenario from recurring. The consequences of such reflective practice can be seen to significantly improve empathy, as illustrated by a systematic review conducted by Chen et al. [5]

In conclusion, we strongly encourage medical schools to strive to incorporate DM into the curriculum through the potential use of SCR and reflective essays. It is also critical that such training is delivered early on in our education. This is important as the study by Ardenghi et al. depicted older students having greater levels of emotional detachment from their negative thoughts compared to younger students, potentially influencing their empathetic skills [1]. Similar studies have also shown a significant decline in empathy as students progress through medical school [3]. This is worrying and outlines the crucial need for DM training earlier on in our studies. Moreover, it is important to highlight to other medical students of its ultimate aim in improving physician well-being and patient care. The pilot SCR that occurred in UCL found that only 64% of students agreed to incorporate SCR into the course despite the fact that 80% said they would attend future rounds [3]. Therefore, the importance of mindfulness training is understood yet the value of it is underestimated by medical students. Medical schools should not only integrate DM into their education programme but also signify its importance to students in their training to become more empathetic and compassionate doctors.
